# Functionality of Fatty Acid Chemoreception: A Potential Factor in the Development of Obesity?

**DOI:** 10.3390/nu5041287

**Published:** 2013-04-17

**Authors:** Lisa Newman, Rivkeh Haryono, Russell Keast

**Affiliations:** Centre for Physical Activity and Nutrition Research, School of Exercise and Nutrition Sciences, Deakin University, Burwood, Victoria 3125, Australia; E-Mails: lpne@deakin.edu.au (L.N.); r.haryono@deakin.edu.au (R.H.)

**Keywords:** oral fatty acid chemoreception, oral sensitivity, fat regulation, obesity, BMI, CD36, GPCRs

## Abstract

Excess dietary fat consumption is recognized as a strong contributing factor in the development of overweight and obesity. Understanding why some individuals are better than others at regulating fat intake will become increasingly important and emerging associative evidence implicates attenuated fatty acid sensing in both the oral cavity and gastrointestinal (GI) tract in the development of obesity. Functional implications of impaired fatty acid chemoreception include diminished activation of the gustatory system, the cephalic response and satiety. This review will focus on knowledge from animal and human studies supporting the existence of oral fatty acid chemoreception including putative oral detection mechanisms, and how sensitivity to fatty acids is associated with fat consumption and fatty food preference.

## 1. Introduction

Obesity is one of the leading causes of preventable disease contributing to negative health outcomes including cardiovascular disease, type-2 diabetes and cancer [1,2]. It is thought that one of the main contributors to overweight and obesity is excess energy consumption, particularly dietary fat. Dietary fat is consumed in excess due to a number of factors including preference for fats, high palatability and satiety responses [3]. One possible mechanism involved in energy intake regulation is the ability to detect fats and other nutrients during ingestion and digestion. When fats are consumed, they are detected by specific receptors in both the mouth and GI tract and induce the release of specific hormones which slow gastric emptying and suppress energy intake [4,5]. Detection of nutrients, in particular fatty acids along the alimentary canal, can directly affect energy intake which raises the possibility that abnormalities to these nutrient detection mechanisms may be associated with excess energy, and possibly fat intake, conceivably promoting obesity [6,7,8,9].

## 2. Overweight/Obesity

The global rise in overweight and obesity is a worldwide health concern and in some regions, has taken the lead over tobacco as the largest preventable cause of disease burden [10]. There have been some reports that overweight and obesity in children is plateauing in some populations, while others predict that in the coming decades increases in the prevalence of obesity will continue, enhancing the burden of obesity-related mortality and morbidity [1,11]. 

There are many factors which contribute to weight gain and the consequential increase in the prevalence of overweight and obesity, including the wide availability of cheap, energy-dense foods [1]. These types of foods are generally high in fat, especially saturated fats, and overconsumption of these foods has been linked to weight gain. Although we have a dietary requirement to ingest fat for many purposes including the requirement of essential fatty acids and the absorption of fat soluble vitamins, the modern day food supply is different from our hunter gatherer heritage when energy dense foods were scarce [12]. At a population level, an excess consumption of dietary fat is one of multiple causal factors in the development of overweight and obesity [13]. Emerging evidence now suggests that dietary fat consumption may be partially regulated by an oral detection mechanism and understanding the functional role of the taste system may be an important factor in understanding reasons for excess energy intake.

## 3. The Sense of Taste and Its Function

Taste is a sense that utilizes chemoreception to detect non-volatile chemicals in potential foods [14]. It is hypothesized that we evolved oral nutrient-toxin detectors (the taste system) to ensure we consume essential nutrients (sugars, fats, amino acids and salts) which are required for functioning and survival, while rejecting foods that may cause harm [12]. Taste qualities including sweet, salty and umami are associated with appetitive responses which, from an evolutionary perspective maximized the chance for consumption of essential nutrients, while aversive responses to excessive sour and bitter tastants maximized the chance of rejection of those foods which may have caused harm [15]. These affective responses to foods partially drive food consumption and individual variation in affective response may influence overconsumption of foods and be a factor in the development of diet-related disease such as obesity. Of importance in this debate is emerging evidence indicating a sixth taste quality responsive to fats, a key macronutrient linked with obesity. 

A taste quality is experienced when the concentration in the oral cavity reaches a level that activates a receptor, which in turn elicits a perception [16]. For example, a compound like sucrose may be in an aqueous solution but at a concentration that cannot be detected. As the concentration of the sucrose increases, the aqueous solution can be discriminated from water and a detection threshold is reached [16]. As the concentration increases further, the recognition threshold will be reached whereby the quality (sweet) will be identified [16]. 

Within the mouth, three types of cells are believed to express taste receptors. The first type are Type I cells (glial-like cells) which express GLAST, a glutamate transporter, NTDPase2, a plasma membrane bound nucleotidase that hydrolyses ATP, ROMK, a K^+^ channel which may be involved in taste cell homeostasis, and ionic currents which may be involved in the perception of salty taste [14]. The next type are Type II (sensory receptor cells) and these cells house the G-Protein Coupled Receptors (GPCRs) which mediate sweet (T1Rs), umami (T1Rs), bitter (T2Rs) and the downstream signaling molecules (α-gustducin, PLCβ2), as well as K^+ ^and Na^+^ channels [14]. Lastly, Type III (pre-synaptic cells) are suspected to form synaptic junctions with nerve terminals and express a number of neuronal like genes, some of which are involved in sour taste perception [14].

## 4. Possibility of Oral Fatty Acid Chemoreception: But Fat Taste?

Emerging evidence in both animals and humans suggests the existence of oral fatty acid chemoreception mediated via receptors located on taste cells [17]. Taste in the traditional sense arguably requires an effective class of stimuli, a taste cell specific transduction mechanism, activation of gustatory nerves by a peripheral taste mechanism, and be perceptually distinguishable from other taste stimuli [18]. Fatty acid most probably satisfies three of the four criteria, but appears to have no discernible quality (*i.e.*, sweet) associated with it. It may be that the taste system has receptors for compounds such as fatty acids, yet the functional response is not a perception, but rather signaling physiologic processes regarding nutrient uptake or toxin expulsion independent of a perception. In this way the perceptual taste system may be a subset of a larger oral chemoreception system that responds to a wider selection of compounds than historically thought. Viewing the sense of taste as a component of a larger inter-related system including chemesthesis, has previously been postulated by Gibson [19] and later extended upon by Green [20]. What follows below is a review of evidence for oral fatty acid chemoreception.

### 4.1. Animal Evidence for Fatty Acid Chemoreception

Animal electrophysiological and behavioral studies have provided evidence in support of an oral chemoreception for fatty acids [21,22]. Gilbertson investigated the effect of different PUFA on K^+^ channels directly on the tongues of rats and found that when exposed to linoleic, linolenic, arachadonic, eicosapentanoic and docosahexanoic fatty acids, inhibition of the K^+^ channel occurs [23]. However, when treated with short-chain fatty acids, no change in K^+^ channels was seen, raising the possibility that multiple fatty acid receptor systems may exist in the oral cavity and that stimulation of the taste cells is selective depending on chain length and saturation of the fatty acid [24]. Supporting the contention of multiple fatty acid receptors in the oral cavity, recent research in rats has identified CD36, GPCR120 and GPCR40 on taste tissue [25,26,27,28,29,30,31,32,33,34,35,36]. Behavioral studies have been conducted using two-bottle preference tests and have established that healthy rodents show a preference for long-chain PUFA when compared to sensory matched oils, even when they are anosmic, sham-fed and potential confounding factors have been removed including, texture, odor and post-ingestive effects [22,37,38,39]. This suggests that there may be an independent oral mechanism for the detection of fatty acids. In addition, rats that were classified as orally hypersensitive to fatty acids consumed less dietary fat and gained less weight when exposed to a high fat diet, whereas orally hyposensitive rats consumed excess fat and rapidly gained weight when fed a high fat diet [21,24]. These studies suggests that oral sensitivity to fatty acids may play a role or be a contributing factor to weight gain in animals.

### 4.2. Human Evidence for Fatty Acid Chemoreception

In humans, several well-controlled studies have been conducted investigating oral detection thresholds for unoxidized fatty acids using sensory matched samples. Mattes found that humans could detect linoleic acid (C18:2), stearic acid (C18:0), lauric acid (C12:0) and caproic acid (C6:0) in the oral cavity in a water emulsion at threshold concentrations ranging from 0.007% (w/v) to 0.06% (w/v) [40,41,42]. Similarly, oral detection thresholds have been found for oleic acid (C18:1), C18:2 and C12:0 when using a stable milk emulsion [43,44,45]. In both studies, non-taste cues were controlled including (1) textural cues, for example, viscosity, which are normally associated with the mouth feel of fats by the use of mineral oils and gums, (2) olfactory cues through the use of nose clips, and (3) visual cues as all tests were conducted under red lights [40,43]. 

Additional studies have found that oral exposure specifically to fat, but not fat mimetics enhances post prandial triglyceride concentrations [46]. This finding followed sham feeding (sample mastication, but not expectorating) of butter and various fat replacers. Furthermore, physiological responses to oral fat exposure include gastric lipase secretion, altered GI transit, pancreatic exocrine secretions, gut hormone release, mobilization of stored lipids from enterocytes, pancreatic endocrine secretion and altered lipoprotein lipase activity [47]. Results support the phenomenon of oral fatty acid chemoreception as fat specific enzymes and other digestive mechanisms throughout the GI tract were initiated when fats were exposed to the oral receptors; however, no such physiological processes were initiated when protein and carbohydrate based fat mimetics were used [46]. It was believed this was due to the cephalic phase response, which involves the release of pre-absorptive enzymes and hormones when a food is tasted. This mechanism is thought to optimize nutrient digestion, absorption and metabolism [48]. 

Recently, oral fatty acid sensitivity has been measured using a novel method whereby subjects “tasted” edible strips, rather than the previous method of liquid emulsions [49]. Although this method is limited by the solubility of fatty acids due to their hydrophobic nature, in the future it may provide a new delivery method which may allow the direct measure of the taste component [49].

Nonetheless, it is unknown if an individual’s fatty acid taste sensitivity as measured by oral detection thresholds remains stable over time, or if the threshold changes as dietary fat varies. Studies have confirmed the reliability and reproducibility of taste thresholds for the five prototypical tastes [50] and our laboratory recently completed similar testing with oral fatty acid thresholds and found results comparable to the prototypical tastes [51].

## 5. Putative Mechanisms for Fatty Acid Chemoreception

**Figure 1:**
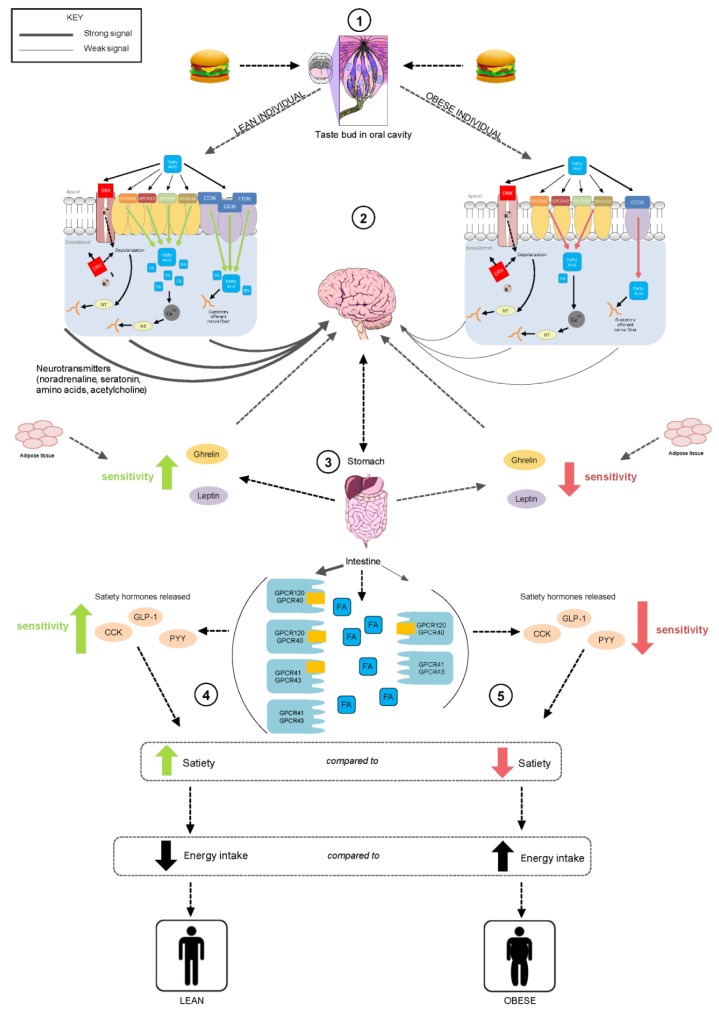
Schematic representation of fatty acid chemoreception in the oral cavity and gastrointestinal tract (alimentary canal) in lean (left) and obese (right) individuals. (**1**) Fat is present in foods in the form of triglycerides; free fatty acids are generated during the breakdown of fats and by lipase enzymes in the oral cavity. (**2**) Fatty acids access putative receptors (CD36, GPCR 40, 41, 43, 120 and delayed rectify potassium (DRK) channels) within taste cells; lean individuals have greater quantities of these receptors, compared to obese individuals. The receptors elicit the release of intracellular Ca^2+^ which in turn activates neurotransmitters and hormones associated with the cephalic response. (**3**) Following fat ingestion, gastric and pancreatic lipase plays a further role in the hydrolysis of fats enabling access to fatty acid receptors on enteroendocrine cells, stimulating satiety hormones and uptake of fatty acids. As a consequence, sensitivity to ghrelin, responsible for hunger stimulation is inhibited, while the satiety inducing hormone leptin is released as are the hormones CCK, PYY, GLP-1. (**4**) In a lean individual, expression of fatty acid receptors is greater therefore increasing fat sensing ability through the alimentary canal, thereby decreasing energy intake. (**5**) In comparison, obese individuals have decreased expression of fatty acid receptors, attenuating fat sensing ability and increasing energy intake.

It is thought that the ability to detect fatty acids is via oral receptors, transporters (CD36, (homologous to Fatty Acid Transporter (FAT) in animals), GPCRs, ion channels (Delayed Rectify Potassium (DRK) channels) and enzymes (lingual lipase) which have been located in the oral cavity on taste receptor cells within the circumvallate and fungiform papillae (Figure 1) [17].

### 5.1. CD36 Transporter and Fatty Acid Transporter (FAT)

One of the proposed mechanisms of oral fatty acid nutrient detection is via CD36, a FAT [25]. CD36 is found in the oral cavity on human taste buds, specifically circumvallate and foliate papillae [26]. Results from a mouse study have shown that inactivating the CD36 receptor eliminated a preference for long chain fatty acid (LCFA) enriched solutions and solid foods [36]. Furthermore, high fat induced rats showed reduced expression of CD36 which may be associated with fatty acid taste adaptation [28]. There is also the possibility that CD36 may be involved with the onset of fat induced satiety [29]. This suggests that the *CD36* receptor plays a direct role in fat perception, and possibly food regulation [30,36].

A recent study using obese humans investigated whether oral sensitivity to fatty acids is attenuated when expression of CD36 is reduced [30]. Subjects were grouped based on whether or not they were a carrier of the variant rs 1761667-A allele of CD36, which has previously been associated with fat metabolism. Subjects were then assessed for their ability to detect C18:1 in water solutions and those who had the genetic variant of CD36 had lower detection thresholds than those without the variant [30]. The same study also investigated thresholds using both a fatty acid and a triglyceride with and without the addition of orlistat and it was found that the inhibition of lipase meant that the release of fatty acids from triglyceride was reduced, therefore attenuating oral fatty acid sensitivity [30]. This study provides strong evidence to suggest CD36 as an orosensory receptor for dietary fatty acids in humans. Additionally, Keller *et al.* [52], has suggested a possible association between polymorphisms in the *CD36* receptor, oral fat perception and fat preference in human subjects. These types of physiological signals which detect fat, and control consumption are plausible and these mechanisms should be explored further.

### 5.2. GPCRs

The possibility that GPCRs may be involved in fatty acid detection in the oral cavity has been suggested and it is thought that CD36 may work together with other possible receptors like GPCRs in a signaling cascade to detect fatty acids [31]. GPCR120 and GPCR40 bind to fatty acids which activate G proteins that cause a release of calcium. This rise in calcium activates the cation channel Transient Receptor Potential channel type M5 (TRPM5), and perception occurs [53]. GPCR120 and GPCR40 have been expressed in the apical portion of type I and II cells from animal taste buds [32,33,54] and more recently, human taste buds [31]. GPCR120 has been isolated in rat circumvallate, fungiform and foliate papillae [34,54], while GPCR40 are expressed specifically in the circumvallate papillae [55]. When wild mice were compared to GPCR120 and GPCR40 knock-out mice, the latter showed an attenuated preference for linoleic acid and C18:1, suggesting these receptors play a role in the perception of fatty acids [32]. Furthermore, when GPCR120 deficient mice were fed a high fat diet, they developed obesity and other side effects of metabolic syndrome indicating a role in regulation of energy intake [35].

### 5.3. DRK Channels

DRK channels are known to be implicated in the transduction pathway of a variety of taste stimuli. A study by Gilbertson [24] found that PUFA slow down DRK polarization on the foliate and circumvallate papillae taste cells and therefore allow fat to be detected. 

### 5.4. Lingual Lipase

The breakdown products of carbohydrates are sugars and proteins are amino acids both of which have taste activity [14]. Therefore, it could be expected that free fatty acids, the breakdown products of triglyceride would have taste activity too. In animals, lingual lipase is released to cleave triglyceride to from fatty acids [27]. Triglyceride are too large to be detected or pass through the cell membrane, whereas free fatty acids are able to translocate through a cellular membrane with ease however, the availability of these in the food matrix is low with fatty foods (including nuts and oils) thought to contain between 0.01% and 0.1% nonesterified fatty acids (NEFA) [56,57]. Despite this, it is thought only small quantities of free fatty acids are required to induce a taste response. Consequently, lipase enzymes are very important as they break the triglyceride down so that free fatty acids can be transduced by cellular pathways [27]. Kawai and Fushiki [27] reported that inhibition of lingual lipase in mice, reduces their preference for lipids greatly. This illustrates that in animal models, lingual lipase plays a significant role in oral fat transduction and perception [27]. In humans however, lingual lipase presence and potential role is debatable. Data has suggested lipolytic activity may be present in humans, however compared to animal models, activity appears to be weak [43] and it is yet to be resolved whether sufficient concentrations of lingual lipase are produced or whether this originates from endogenous sources or otherwise. Kulkarni and Mattes [57] have recently observed mastication of fatty foods (almond butter almonds, olive oil and shredded coconut) can increase salivary NEFA concentrations from between 20 and 60 µM compared to control stimulated saliva levels, suggesting these concentrations in foods may be enough to initiate gustatory signaling.

There are multiple putative mechanisms to initiate oral fatty acid detectors including CD36 transporter, FAT, GPCRs, DRK channels and lingual lipase hydrolysis of fats to fatty acids; however it is yet to be elucidated if/how these function independently or together in oral fatty acid detection.

## 6. Possible Functions of Fatty Acid Chemoreception

Recent research has implicated oral fatty acid sensitivity in weight gain, with those less sensitive (higher taste threshold) having a higher body mass index (BMI) [43,44]. Oral fatty acid sensitivity refers to an individual’s ability to detect fatty acids in a complex matrix when tasting a solution. The ability to detect fatty acids differs between individuals and variance is likely a result of oral peripheral mechanisms responsible for chemoreception, such as differences in fatty acid receptor functionality or papillae density [58]. Indeed, a positive association between sensitivity and number of taste papillae has been reported [21,59]. Selected studies have found a link between sensitivity to and liking of, or dislike to certain tastants, however a myriad of factors presumably drive this association [60,61,62]. Thus, further research is required to elucidate whether differences in oral chemoreception (taste sensitivity) affect food choice, preferences and taste receptor expression, or *vice versa*.

Kamphuis *et al.* [63] investigated whether there was a link between oral fatty acid sensitivity and BMI. The authors suggested the existence of “fat-tasters” and “fat non-tasters” reporting that “fat-tasters” had a lower BMI than “fat non-tasters” however, there was no link found between oral fatty acid sensitivity and fat consumption [24,63]. Nevertheless, given the low concentration (0.0028% weight/volume (w/v) C18:2) and the fact that only one type of fat was used to determine fat taster status, the existence of fat non-tasters remains controversial. Conversely, a relationship between fat intake and obesity may exist as fat intake is high in the obese population.

The relationship between oral fatty acid sensitivity, BMI and dietary fat intake has recently been investigated by our group [43]. It was found that those who were more sensitive to the fatty acid C18:1 had lower energy intakes and consumed less total dietary fats and saturated fats and were also better at detecting the fat content of food (custard) [43]. This suggests that oral fatty acid chemoreception may be used for the identification of fats. Additionally, individuals who were classified as hypersensitive also had lower BMIs than hyposensitive individuals [43]. Another study by Stewart *et al.* [44] extended these results and also found a relationship in humans between fatty acid sensitivity, food consumption and dietary behaviors, whereby those who were hyposensitive consumed more high fat dairy products, high fat spreads, and fatty red meat. Conversely, hypersensitive individuals reported behaviors including trimming the fat off meat and avoiding saturated fats [44]. The exact mechanisms responsible for these differences between hyper- and hyposensitive groups are not yet understood. While fatty acid sensitivity may drive fat consumption and preference, the reverse may also be true in that prolonged fat consumption and/or preference may be a predictor of sensitivity. In this paradigm, recent research suggests that similarly to the oral cavity, GI responses are attenuated.

## 7. Fatty Acid Sensitivity in the Oral Cavity and GI Tract

An important mechanism involved in energy intake regulation is the body’s ability to detect fat and other nutrients in the oral cavity and GI tract. Evidence now exists indicating that sweet and umami taste receptors for sugars (carbohydrates) and amino acids (proteins) respectively, are co-located in the GI tract [14], which provides foundation evidence for the hypothesis that the taste system is the first contact for a coordinated alimentary canal nutrient detection system. It is now thought that the same relationship exists for fats, with fatty acid detection occurring in both the oral cavity and GI tract (Figure 1).

During digestion, fats have potent effects on hormones that regulate food intake, for example, CCK and glucagon-like peptide-1 (GLP-1) decrease gastric emptying and secretion of the hunger stimulating hormone ghrelin, while leptin binds to neuropeptide Y (NPY) to reduce appetite and induce satiety in a lean individual [4,64]. Studies have also suggested that these hormone responses are impaired in obese individuals, raising the possibility that fat intake may be poorly controlled in the obese population due to a dysfunction in appetite regulation (Figure 1). When comparing lean and obese individuals after an introduodenal infusion of C18:1, obese individuals had a reduced stimulation of pyloric motility, thus, they were less able to sense fatty acids along the GI tract [65]. Additionally, oral fatty acid sensitivity was also impaired in the obese suggesting a coordinated alimentary canal response to fatty acids.

## 8. Dietary Influences on Fatty Acid Chemoreception

Consumption of a high fat diet has been shown to decrease oral fatty acid sensitivity [66]. Dietary influences on the plasticity of fatty acid, specifically C18:1, detection thresholds was investigated whereby subjects were asked to consume a low fat diet (<20% fat) for 4 weeks and after a 2-week wash out period, then followed a high fat diet for 4 weeks (>40%). After the low fat diet both lean and obese subjects’ C18:1 detection thresholds decreased, in other words their sensitivity increased, and after the high fat diet thresholds increased, however, this only occurred in the lean subjects with no change in thresholds for the obese population [66]. This suggests that in the obese population consumption of a high fat diet has occurred that has promoted habituation which therefore decreased the physiological and psychological effects of fat, which in turn decreased one’s oral sensitivity to fat and possibly promoted obesity.

## 9. Environmental Influences on Gene Expression

As previously mentioned, fats are thought to be detected by fatty acid specific receptors in both the oral cavity and GI tract. The role of these receptors in fat detection is now becoming more apparent with studies highlighting dietary influences on receptor expression. While there is a paucity of evidence in humans, emerging evidence in animals is showing promising findings. Following the consumption of a high fat diet, CD36 receptor expression on the lingual tissue of rats was reduced highlighting the potential link between fat consumption and CD36 receptor expression [28]. While suggestive at this stage, it does appear that over a short period of time, an individual can adapt to a fatty food environment. In doing so, they become less sensitive to the physiological (changes in GI motility, hormone secretion, suppression of appetite) and psychological effects of fat [12], thus attenuating the body’s response to the stimuli. Whether this is a result of changes in gene expression, or perhaps reduced receptor sensitivity remains unresolved. Humans may quickly adapt to the new environment by consuming more fats, but in doing so, the prevalence of obesity is increasing as we have not yet developed a way to deal with the excess fat being consumed. 

## 10. Conclusions

In summary, excess fat consumption is a major cause of obesity as well as other diseases including CVD and type-2 diabetes. Fat intake can be influenced by many factors including habituation and satiety mechanisms and an individual’s ability to detect fatty acids in the oral cavity and GI tract via fat specific receptors. There is substantive emerging evidence that an oral nutrient detection system for fatty acids exists in humans. Similarly to the five basic tastes, the ability to detect fatty acids in the oral cavity varies amongst the population. This variance may be a factor in influencing one’s consumption of high-fat foods, with those who are hypersensitive consuming less fat, and preferring low-fat foods than hyposensitive individuals who consume more fat and prefer high-fat foods. It is unknown what the main drivers of this concept are, or if in fact reduced fatty acid sensitivity is a predictor of dietary fat consumption, or *vice versa*. Knowing the reproducibility and stability of an individual’s oral sensitivity is important and needs to be verified. Oral fatty acid sensitivity and its potential links to obesity is a controversial area of research with more investigation needed from a variety of scientific disciplines, but emerging evidence linking oral fatty acid sensitivity with development of obesity is promising. Currently the exact mechanisms associating oral fatty acid sensitivity, fat consumption and weight gain remain largely elusive. Dietary intake may not be the only factor influencing one’s sensitivity; other considerations, for example the expression of taste receptors in the oral cavity are likely to play an influential role. Investigating oral fatty acid sensitivity and its potential links with dietary fat intake and putative fatty acid taste receptor expression will build upon present knowledge; if indeed receptor expression can be modulated in response to a high fat diet, or if expression is a driver of fat preference and consequent consumption may provide more of an insight into reasons for excess fat consumption. These ideas are conjectural, but may identify strategies to reduce obesity and related pathologies.

## References

[1] Swinburn B.A., Sacks G., Hall K.D., McPherson K., Finegood D.T., Moodie M.L., Gortmaker S.L. (2011). The global obesity pandemic: Shaped by global drivers and local environments. Lancet.

[2] Wang Y.C., McPherson K., Marsh T., Gortmaker S.L., Brown M. (2011). Health and economic burden of the projected obesity trends in the USA and the UK. Lancet.

[3] Snoek H.T.M., Huntjens L., van Gemert L.J., de Graaf C., Weenen H. (2004). Sensory-specific satiety in obese and normal-weight women. Am. J. Clin. Nutr..

[4] Feltrin K.L., Little T.J., Meyer J.H., Horowitz M., Smout A.J.P.M., Wishart J., Pilichiewicz A.N., Rades T., Chapman I.M., Feinle-Bisset C. (2004). Effects of intraduodenal fatty acids on appetite, antropyloroduodenal motility, and plasma CCK and GLP-1 in humans vary with their chain length. Am. J. Physiol. Regul. Integr. Comp. Physiol..

[5] Cummings D.E., Overduin J. (2007). Gastrointestinal regulation of food intake. J. Clin. Invest..

[6] Blundell J.E., Macdiarmid J.I. (1997). Fat as a risk factor for overconsumption: Satiation, satiety, and patterns of eating. J. Am. Diet. Assoc..

[7] Rolls B.J., Kim-Harris S., Fischman M.W., Foltin R.W., Moran T.H., Stoner S.A. (1994). Satiety after preloads with different amounts of fat and carbohydrate: Implications for obesity. Am. J. Clin. Nutr..

[8] Speechly D.P., Buffenstein R. (2000). Appetite dysfunction in obese males: Evidence for role of hyperinsulinaemia in passive overconsumption with a high fat diet. Eur. J. Clin. Nutr..

[9] Westerterp K.R. (2006). Perception, passive overfeeding and energy metabolism. Physiol. Behav..

[10] Hoad V., Somerford P., Katzenellenbogen J. (2010). High body mass index overtakes tobacco as the leading independent risk factor contributing to disease burden in Western Australia. Aust. N. Z. J. Public Health.

[11] Rokholm B., Baker J.L., Sørensen T.I.A. (2010). The levelling off of the obesity epidemic since the year 1999—A review of evidence and perspectives. Obes. Rev..

[12] Cordain L., Eaton S.B., Sebastian A., Mann N., Lindeberg S., Watkins B.A., O’Keefe J.H., Brand-Miller J. (2005). Origins and evolution of the Western diet: Health implications for the 21st century. Am. J. Clin. Nutr..

[13] Bray G.A., Paeratakul S., Popkin B.M. (2004). Dietary fat and obesity: A review of animal, clinical and epidemiological studies. Physiol. Behav..

[14] Bachmanov A.A., Beauchamp G.K. (2007). Taste receptor genes. Annu. Rev. Nutr..

[15] Gilbertson T.A., Damak S., Margolskee R.F. (2000). The molecular physiology of taste transduction. Curr. Opin. Neurobiol..

[16] Keast R.S.J., Roper J. (2007). A complex relationship among chemical concentration, detection threshold, and suprathreshold intensity of bitter compounds. Chem. Senses.

[17] Laugerette F., Gaillard D., Passilly-Degrace P., Niot I., Besnard P. (2007). Do we taste fat?. Biochimie.

[18] Mattes R.D. (2011). Accumulating evidence supports a taste component for free fatty acids in humans. Physiol. Behav..

[19] Gibson J.J. (1966). The Senses Considered as Perceptual System.

[20] Green B.G. (2003). Studying taste as a cutaneous sense. Food Qual. Prefer..

[21] Gilbertson T.A., Liu L., Kim I., Burks C.A., Hansen D.R. (2005). Fatty acid responses in taste cells from obesity-prone and -resistant rats. Physiol. Behav..

[22] Takeda M., Sawano S., Imaizumi M., Fushiki T. (2001). Preference for corn oil in olfactory-blocked mice in the conditioned place preference test and the two-bottle choice test. Life Sci..

[23] Gilbertson T.A., Fontenot D.T. (1997). Fatty acid modulation of K+ channels in taste receptor cells: Gustatory cues for dietary fat. Am. J. Physiol..

[24] Gilbertson T.A. (1998). Gustatory mechanisms for the detection of fat. Curr. Opin. Neurobiol..

[25] Abumrad N.A. (2005). CD36 may determine our desire for dietary fats. J. Clin. Invest..

[26] Simons P.J., Kummer J.A., Luiken J.J.F.P., Boon L. (2010). Apical CD36 immunolocalization in human and porcine taste buds from circumvallate and foliate papillae. Acta Histochem..

[27] Kawai T., Fushiki T. (2003). Importance of lipolysis in oral cavity for orosensory detection of fat. Am. J. Physiol. Regul. Integr. Comp. Physiol..

[28] Zhang X.-J., Zhou L.-H., Ban X., Liu D.-X., Jiang W., Liu X.-M. (2011). Decreased expression of CD36 in circumvallate taste buds of high-fat diet induced obese rats. Acta Histochem..

[29] Naville D., Duchampt A., Vigier M., Oursel D., Lessire R., Poirier H., Niot I., Bégeot M., Besnard P., Mithieux G. (2012). Link between intestinal CD36 ligand binding and satiety induced by a high protein diet in mice. PLoS One.

[30] Pepino M.Y., Love-Gregory L., Klein S., Abumrad N.A. (2011). The fatty acid translocase gene, CD36, and lingual lipase influence oral sensitivity to fat in obese subjects. J. Lipid Res..

[31] Galindo M.M., Voigt N., Stein J., van Lengerich J., Raguse J.-D., Hofmann T., Meyerhof W., Behrens M. (2012). G protein-coupled receptors in human fat taste perception. Chem. Senses.

[32] Cartoni C., Yasumatsu K., Ohkuri T., Shigemura N., Yoshida R., Godinot N., le Coutre J., Ninomiya Y., Damak S. (2010). Taste preference for fatty acids is mediated by GPR40 and GPR120. J. Neurosci..

[33] Gotoh C., Hong Y.-H., Iga T., Hishikawa D., Suzuki Y., Song S.-H., Choi K.-C., Adachi T., Hirasawa A., Tsujimoto G. (2007). The regulation of adipogenesis through GPR120. Biochem. Biophys. Res. Commun..

[34] Matsumura S., Eguchi A., Mizushige T., Kitabayashi N., Tsuzuki S., Inoue K., Fushiki T. (2009). Colocalization of GPR120 with phospholipase-Cβ2 and α-gustducin in the taste bud cells in mice. Neurosci. Lett..

[35] Ichimura A., Hirasawa A., Poulain-Godefroy O., Bonnefond A., Hara T., Yengo L., Kimura I., Leloire A., Liu N., Iida K. (2012). Dysfunction of lipid sensor GPR120 leads to obesity in both mouse and human. Nature.

[36] Laugerette F., Passilly-Degrace P., Patris B., Niot I., Febbraio M., Montmayeur J.P., Besnard P. (2005). CD36 involvement in orosensory detection of dietary lipids, spontaneous fat preference, and digestive secretions. J. Clin. Invest..

[37] Tsuruta M., Kawada T., Fukuwatari T., Fushiki T. (1999). The orosensory recognition of long-chain fatty acids in rats. Physiol. Behav..

[38] Fukuwatari T., Shibata K., Iguchi K., Saeki T., Iwata A., Tani K., Sugimoto E., Fushiki T. (2003). Role of gustation in the recognition of oleate and triolein in anosmic rats. Physiol. Behav..

[39] Mindell S., Smith G.P., Greenberg D. (1990). Corn oil and mineral oil stimulate sham feeding in rats. Physiol. Behav..

[40] Mattes R.D. (2009). Oral detection of short-, medium-, and long-chain free fatty acids in humans. Chem. Senses.

[41] Mattes R.D. (2009). Oral thresholds and suprathreshold intensity ratings for free fatty acids on 3 tongue sites in humans: Implications for transduction mechanisms. Chem. Senses.

[42] Mattes R.D. (2009). Brief oral stimulation, but especially oral fat exposure, elevates serum triglycerides in humans. Am. J. Physiol. Gastrointest. Liver Physiol..

[43] Stewart J.E., Feinle-Bisset C., Golding M., Delahunty C., Clifton P.M., Keast R.S.J. (2010). Oral sensitivity to fatty acids, food consumption and BMI in human subjects. Br. J. Nutr..

[44] Stewart J.E., Newman L.P., Keast R.S.J. (2011). Oral sensitivity to oleic acid is associated with fat intake and body mass index. Clin. Nutr..

[45] Stewart J.E., Seimon R.V., Otto B.R., Keast R.S.J., Clifton P.M., Feinle-Bisset C. (2011). Marked differences in gustatory and gastrointestinal sensitivity to oleic acid between lean and obese men. Am. J. Clin. Nutr..

[46] Mattes R.D. (2001). Oral exposure to butter, but not fat replacers elevates postprandial triacylglycerol concentration in humans. J. Nutr..

[47] Mattes R.D. (2005). Fat taste and lipid metabolism in humans. Physiol. Behav..

[48] Crystal S.R., Teff K.L. (2006). Tasting fat: Cephalic phase hormonal responses and food intake in restrained and unrestrained eaters. Physiol. Behav..

[49] Ebba S., Abarintos R.A., Kim D.G., Tiyouh M., Stull J.C., Movalia A., Smutzer G. (2012). The examination of fatty acid taste with edible strips. Physiol. Behav..

[50] Ahne G., Erras A., Hummel T., Kobal G. (2000). Assessment of gustatory function by means of tasting tablets. Laryngoscope.

[51] Newman L.P., Keast R.S.J. (2013). The test retest reliability of fatty acid taste thresholds. Chemosens. Percept..

[52] Keller K.L., Liang L.C.H., Sakimura J., May D., van Belle C., Breen C., Driggin E., Tepper B.J., Lanzano P.C., Deng L. (2012). Common variants in the CD36 gene are associated with oral fat perception, fat preferences, and obesity in African Americans. Obesity (Silver Spring).

[53] Liu P., Shah B.P., Croasdell S., Gilbertson T.A. (2011). Transient receptor potential channel Type M5 is essential for fat taste. J. Neurosci..

[54] Matsumura S., Mizushige T., Yoneda T., Iwanaga T., Tsuzuki S., Inoue K., Fushiki T. (2007). GPR expression in the rat taste bud relating to fatty acid sensing. Biomed. Res..

[55] Covington D., Briscoe C., Brown A., Jayawickreme C. (2006). The G-protein-coupled receptor 40 family (GPR40-GPR43) and its role in nutrient sensing. Biochem. Soc. Trans..

[56] Laffargue A., de Kochko A., Dussert S. (2007). Development of solid-phase extraction and methylation procedures to analyse free fatty acids in lipid-rich seeds. Plant. Physiol. Biochem..

[57] Kulkarni B., Mattes R. (2013). Evidence for presence of nonesterified Fatty acids as potential gustatory signaling molecules in humans. Chem. Senses.

[58] Chale-Rush A., Burgess J.R., Mattes R.D. (2007). Multiple routes of chemosensitivity to free fatty acids in humans. Am. J. Physiol. Gastrointest. Liver Physiol..

[59] Drayna D. (2005). Human taste genetics. Annu. Rev. Genomics Hum. Genet..

[60] Drewnowski A., Henderson S.A., Hann C.S., Berg W.A., Ruffin M.T. (2000). Genetic taste markers and preferences for vegetables and fruit of female breast care patients. J. Am. Diet. Assoc..

[61] Keller K.L., Steinmann L., Nurse R.J., Tepper B.J. (2002). Genetic taste sensitivity to 6-n-propylthiouracil influences food preference and reported intake in preschool children. Appetite.

[62] Turnbull B., Matisoo-Smith E. (2002). Taste sensitivity to 6-*n*-propylthiouracil predicts acceptance of bitter-tasting spinach in 3–6-y-old children. Am. J. Clin. Nutr..

[63] Kamphuis M.M., Westerterp-Plantenga M.S., Saris W.H. (2001). Fat-specific satiety in humans for fat high in linoleic acid *vs* fat high in oleic acid. Eur. J. Clin. Nutr..

[64] Heini A.F., Lara-Castro C., Kirk K.A., Considine R.V., Caro J.F., Weinsier R.L. (1998). Association of leptin and hunger-satiety ratings in obese women. Int. J. Obes. Relat. Metab. Disord..

[65] Little T.J., Feinle-Bisset C. (2010). Oral and gastrointestinal sensing of dietary fat and appetite regulation in humans: Modification by diet and obesity. Front. Neurosci..

[66] Stewart J.E., Keast R.S.J. (2011). Recent fat intake modulates fat taste sensitivity in lean and overweight subjects. Int. J. Obes..

